# Methylmalonic Acid and Homocysteine as Indicators of Vitamin B-12 Deficiency in Cancer

**DOI:** 10.1371/journal.pone.0147843

**Published:** 2016-01-25

**Authors:** Pankaj Vashi, Persis Edwin, Brenten Popiel, Carolyn Lammersfeld, Digant Gupta

**Affiliations:** Cancer Treatment Centers of America^®^ (CTCA) at Midwestern Regional Medical Center, 2520 Elisha Ave, Zion, Illinois, 60099, United States of America; CSIR-INSTITUTE OF GENOMICS AND INTEGRATIVE BIOLOGY, INDIA

## Abstract

**Background/Aims:**

Normal or high serum vitamin B-12 levels can sometimes be seen in a B-12 deficient state, and can therefore be misleading. High levels of Methymalonic Acid (MMA) and Homocysteine (HC) have been identified as better indicators of B-12 deficiency than the actual serum B-12 level itself. We evaluated the prevalence of vitamin B-12 deficiency using appropriate cut-off levels of vitamin B-12, MMA and HC, and determined the relationship between serum levels of vitamin B-12, MMA and HC in cancer.

**Methods:**

This is a cross-sectional study using a consecutive case series of 316 cancer patients first seen at Cancer Treatment Centers of America^®^ (CTCA) at Midwestern Regional Medical Center between April 2014 and June 2014. All patients were evaluated at baseline for vitamin B-12 (pg/mL), MMA (nmol/L) and HC (μmol/L) levels. In accordance with previously published research, the following cut-offs were used to define vitamin B-12 deficiency: <300 pg/mL for vitamin B-12, >260 nmol/L for MMA and >12 μmol/L for HC. The relationship between B-12, MMA and HC was evaluated using Spearman's rho correlation coefficient and cross-tabulation analysis. Receiver Operating Characteristic (ROC) curves were estimated using the non-parametric method to further evaluate the diagnostic accuracy of vitamin B-12 using Fedosov quotient as the "gold standard".

**Results:**

Mean age at presentation was 52.5 years. 134 (42.4%) patients were males while 182 (57.6%) were females. Median vitamin B-12, MMA and HC levels were 582.5 pg/mL, 146.5 nmol/L and 8.4 μmol/L respectively. Of 316 patients, 28 (8.9%) were vitamin B-12 deficient based on vitamin B-12 (<300pg/mL), 34 (10.8%) were deficient based on MMA (>260 nmol/L) while 55 (17.4%) were deficient based on HC (>12 μmol/L). Correlation analysis revealed a significant weak negative correlation between vitamin B-12 and MMA (rho = -0.22) as well as B-12 and HC (rho = -0.35). ROC curves suggested MMA to have the best discriminatory power in predicting B-12 deficiency.

**Conclusion:**

Vitamin B-12 is poorly correlated with MMA and HC in cancer. Using serum vitamin B-12 alone to evaluate B-12 status in cancer may fail to identify those with functional deficiency. A thorough clinical assessment is important to identify patients that may have risk factors and/or symptoms suggestive of deficiency. These patients should have additional testing of MMA and HC regardless of their B-12 levels.

## Introduction

Vitamin B-12 is a water soluble vitamin. The main sources of vitamin B-12 are animal foods including meats and dairy products, as well as foods fortified with the vitamin. Vitamin B-12 plays a role in neurologic function and is necessary for maintaining nerve sheaths as well as function of the nerves [1]. In the absence of adequate vitamin B-12, nerves can be damaged and nerve function compromised. Deficiency is associated with megaloblastic anemia and several neurologic manifestations such as paresthesias, peripheral neuropathy, and demyelination of the corticospinal tract and dorsal columns [1; 2].

Deficiencies of vitamin B-12 can arise from nutritional factors, malabsorption and other gastrointestinal (GI) causes [3]. The elderly and alcoholics are prone to deficiency due to poor oral intake. Also, strict vegans can be at risk if they do not consume fortified foods or an alternate source of the nutrient. Malabsorption can arise from impairment in gastric acid secretion, including gastrectomy as well as enteritis and resection of the ileum. Medications including proton pump inhibitors, H2 receptor antagonists, and biguanides (metformin) can also contribute to malabsorption and deficiency [1]. Finally, vitamin B-12 deficiency is seen in patients with pernicious anemia due to lack of intrinsic factor in the stomach.

Cancer patients are also vulnerable to vitamin B-12 deficiency due to poor oral intake, malabsorption, GI surgeries, medications and enteritis. Vitamin B-12 deficiency in cancer patients has been identified as a predisposing condition that may increase the risk of developing chemotherapy-induced peripheral neuropathy (CIPN) [4]. CIPN is one of the most common non-hematological adverse effects of many chemotherapy regimens such as taxanes, platinum compounds, vinca alkaloids, proteasome inhibitors and 5-fluorouracil [2;4–6]. CIPN can be dose-limiting, and significantly impact quality of life, because it can continue and even worsen after the completion of chemotherapy. Vitamin B-12 deficiency may also develop during chemotherapy administration and can potentially predispose patients to developing CIPN [2]. Moreover, the neurotoxic effects of chemotherapy can be compounded by a pre-existing vitamin B-12 deficiency. Consequently, diagnosing vitamin B-12 deficiency early in the natural history of cancer can help identify patients who are at a greater likelihood of subsequently developing CIPN.

However, vitamin B-12 deficiency can be difficult to diagnose. Diagnosis is typically based on measurement of serum vitamin B-12 levels (usually less than 200 pg/mL), however, about 50 percent of patients with subclinical disease have normal vitamin B-12 levels [1;3]. Moreover, elevated serum levels of vitamin B-12 can be accompanied by signs of deficiency as well, suggesting a functional deficiency from defects in tissue uptake and action of vitamin B-12 at the cellular level. Abnormally high serum vitamin B-12 levels can be caused by solid cancers, hematologic malignancies, liver and kidney disease. Due to the lack of a clear association between serum vitamin B-12 and its deficiency, it has been proposed that functional vitamin B-12 deficiency can occur at any serum level [7;8]. As a result, more sensitive methods of screening for vitamin B-12 deficiency are needed. One such method utilizes the measurement of metabolites that accumulate as a result of vitamin B-12 deficiency.

There are two enzymatic reactions that are dependent on vitamin B-12. Vitamin B-12 is required for methylmalonic acid (MMA) to be converted to succinyl-CoA, and in combination with folic acid, for homocysteine (HC) to be converted to methionine [9]. Therefore, MMA is clearly more specific to vitamin B-12 deficiency compared to HC. A deficiency of vitamin B-12 at the tissue level can lead to elevation of both MMA and HC [1] even when serum vitamin B-12 concentrations are within the reference values. Elevated MMA and HC levels together have been found to be 99.8% sensitive for diagnosing functional vitamin B-12 deficiency [10], which is defined as elevated MMA and HC levels despite normal vitamin B-12 levels in asymptomatic individuals [11–13]. Therefore, using serum vitamin B-12 testing alone may under-diagnose the deficiency of this vitamin.

MMA and HC have been identified as early markers of vitamin B-12 deficiency in different subjects such as general population [14], elderly [15–17], Asian Indians [18], infants [19], pregnant women [20], healthy women [21], type 2 diabetes [22], phenylketonuria [13] and renal insufficiency [12], however, no studies conducted till date have evaluated the interrelationships between vitamin B-12, MMA and HC in cancer patients. In non-cancerous conditions, vitamin B-12 testing is usually done in highly suspicious clinical conditions like megaloblastic anemia, unexplained neuropathy and atrophic gastritis with or without pernicious anemia. Unfortunately, anemia and some form of neuropathy are very common in patients with solid tumors and thus it is difficult to suspect vitamin B-12 deficiency in these patients. Therefore the aims of the present study were to determine the following in a cohort of cancer patients treated at a tertiary care cancer center: 1) the prevalence of vitamin B-12 deficiency using appropriate cut-off levels of vitamin B-12, MMA and HC; 2) the relationship between serum vitamin B-12, MMA and HC.

## Methods

### Study Design and Patient Population

This is a cross-sectional study using a consecutive case series of 316 cancer patients first seen at Cancer Treatment Centers of America^®^ (CTCA) at Midwestern Regional Medical Center between April 2014 and June 2014. All adult cancer patients coming to CTCA for treatment during the three-month study period were considered eligible irrespective of their age, gender, cancer diagnosis or any other clinical or demographic characteristic. The only inclusion criterion was a diagnosis of cancer in patients greater than 18 years of age. There were no exclusion criteria. We included a consecutive case series of patients to avoid non-response and minimize the probability of selection bias. All eligible patients were identified from the hospital’s tumor registry.

This study was approved by the Institutional Review Board (IRB) at Cancer Treatment Centers of America^®^. The need for informed consent was waived by the IRB because there was no direct patient contact in this study. This study involved collection of existing data from patient records in such a manner that subjects cannot be identified, directly or through identifiers linked to the subjects. Patient records/information was anonymized and de-identified prior to analysis.

### Biochemical Methods

Serum vitamin B-12 levels were measured using the ARCHITECT B-12 assay is a two-step assay with an automated sample pretreatment, for determining the presence of B-12 in human serum using chemiluminescent microparticle immunoassay technology with flexible assay protocols, referred to as Chemiflex. MMA was measured using Liquid chromatography/tandem mass spectrometry (LC/MS-MS). HC was determined using the Dimension Vista^®^ System where bound homocysteine in the sample is reduced to free homocysteine by the action of dithiothreitol, and then converted enzymatically to S-adenosylhomocysteine (SAH).

Conjugated S-adenosylcysteine (SAC), added at the onset of the reaction, competes with the SAH in the sample for bonding by anti-SAH antibodies bound to polystyrene particles. In the presence of SAH, there is either no aggregation or a weak aggregation of particles. In the absence of SAH in the sample, an aggregation of the polystyrene particles by the conjugated SAC occurs. The higher the SAH content of the reaction mixture, the smaller the scattered light. B-12, MMA and HC were determined simultaneously in each patient.

### Statistical Analysis

Distributions of vitamin B-12, MMA and HC were assessed for normality using a combination of Shapiro–Wilk test, histogram and normal Q-Q plot. Because of highly skewed distributions for all, non-parametric Mann-Whitney and Kruskal–Wallis tests were used for comparison of these parameters across different patient groups, as appropriate. The correlation between vitamin B-12, MMA and HC was evaluated using Spearman's rho correlation coefficient in the total population as well as stratified by certain clinical and demographic characteristics. Vitamin B-12, MMA and HC were also analyzed as categorical variables. In accordance with previously published research, the following cut-offs were used to define vitamin B-12 deficiency: <300 pg/mL for vitamin B-12, >260 nmol/L for MMA and >12 μmol/L for HC [8;14]. The relationships between these categorical vitamin B-12, MMA and HC variables were evaluated using the chi-square test.

Receiver Operating Characteristic (ROC) curves were estimated using the non-parametric method [23;24] to further evaluate the diagnostic accuracy of vitamin B-12. We used a combined indicator of vitamin B-12 status (cB12) as the “gold-standard” for ROC analysis. The cB12 is a novel approach validated by Fedosov et al. that combines four B-12 biomarkers [B-12, MMA, HC and holotranscobalamin (holoTC)] into one combined marker (cB12) to increase the precision of diagnosis [25;26]. The cB12 can also be calculated when one or two biomarkers are missing, as described in the study by Fedosov et al. [25]. In our study, holoTC data were not available and therefore the calculation of cB12 was done using B-12, MMA and HC. Consistent with previous research in this area, a cB12 value of < −0.5 point was used to indicate vitamin B-12 deficiency [25;26]. The area under the curve (AUC) was calculated to determine the accuracy of serum vitamin B-12 in predicting cB12. The further the curve lies above the reference line, the more accurate the test. Coordinates of the curve were examined across the full range of potential vitamin B-12 cut-off values in an attempt to select an optimal cut-off that properly balanced the needs of sensitivity and specificity. Since smaller values of vitamin B-12 are believed to indicate worsening of deficiency, sensitivity was defined as the proportion of B-12 deficient patients (as defined by cB12 values < −0.5) with vitamin B-12 results smaller than the cut-off (i.e. true positives). Similarly, specificity was defined as the proportion of B-12 sufficient patients (as defined by cB12 values > = −0.5) with vitamin B-12 results greater than equal to the cut-off (i.e. true negatives). For the purpose of ROC analysis, patients with increased serum creatinine (>1.2 mg/dl), patients who were malnourished and patients with estimated glomerular filtration rate (eGFR) <60 ml/min/1.73m^2^ were excluded from the ROC analyses in order to reduce potential confounding by eGFR. eGFR was calculated according to the chronic kidney disease epidemiology collaboration (CKD-EPI) formula [27].

No formal sample size calculations were conducted for this analysis. All data were analyzed using IBM SPSS version 23.0 (IBM, Armonk, NY, USA). All analyses were two-tailed, and a difference was considered to be statistically significant if the p value was < = 0.05.

## Results

### Patient Characteristics

A total of 316 adult cancer patients came to our institution between April 2014 and June 2014. All of them were examined for eligibility and found to be eligible. As a result, all 316 patients were included in the analysis with no patient exclusions at any stage. **Table 1**displays the baseline characteristics of our patients. The majority of the patients were newly-diagnosed (71.5%), non-diabetic (85.4%), well-nourished (77.5%), had normal serum creatinine (94.3%) and normal eGFR (93%). The most common cancers were breast (24.7%), colorectal (17.4%), lung (14.2%), prostate (7.9%) and pancreas (6.6%).

**Table 1:** Baseline Patient Characteristics.

Characteristic	Categories	Number (Percent)
Age in years	Mean	52.5
	Median	54
	Range	22–82
Prior Treatment History	Previously-treated	90 (28.5)
	Newly-diagnosed	226 (71.5)
Gender	Male	134 (42.4)
	Female	182 (57.6)
Cancer Type	Breast	78 (24.7)
	Colorectal	55 (17.4)
	Lung	45 (14.2)
	Prostate	25 (7.9)
	Pancreas	21 (6.6)
	Others	92 (29.1)
Diabetes	Yes	46 (14.6)
	No	270 (85.4)
Serum Creatinine	High (>1.2 mg/dl)	18 (5.7)
	Normal (< = 1.2 mg/dl)	298 (94.3)
eGFR	Low (<60 ml/min/1.73m^2^)	22 (7)
	Normal (> = 60 ml/min/1.73m^2^)	294 (93)
Subjective Global Assessment	Malnourished	71 (22.5)
	Well-nourished	245 (77.5)

(mg/dl = milligrams per deciliter; eGFR = estimated glomerular filtration rate).

### Distribution of Vitamin B-12, MMA and HC

Median serum vitamin B-12, MMA and HC levels were 582.5 pg/mL, 146.5 nmol/L and 8.4 μmol/L respectively. **Table 2**provides the distribution of vitamin B-12, MMA and HC levels across different categories of clinical and demographic characteristics. Males had significantly worse levels (lower vitamin B-12 and higher MMA and HC) for all 3 parameters compared to females. Similarly, patients with high serum creatinine and low eGFR had significantly worse levels for all 3 parameters compared to those with normal renal function. Malnourished patients had significantly worse MMA and HC levels compared to well-nourished patients. Patients older than 54 years (median) had significantly worse MMA and HC levels compared to patients younger than 54 years. Finally, patients with colorectal, prostate and pancreatic cancers had significantly worse levels for all 3 parameters compared to those with breast and lung cancers.

**Table 2:** Distribution of Vitamin B-12, MMA and HC Scores.

Characteristic	Vitamin B-12	MMA	HC
	(pg/mL)	(nmol/L)	(μmol/L)
	Median (range)	Median (range)	Median (range)
Total Population	582.5 (149–2000)	146.5 (50–1404)	8.4 (3.7–61)
Age			
< Median of 54 years	586 (152–2000)	135.5 (50–1130)	7.9 (3.7–61)
> = Median of 54 years	572.5 (149–2000)	165.5 (50–1404)	8.8 (3.7–21.9)
	*p = 0*.*63*	*p<0*.001*	*p = 0*.002*
Gender			
Male	535.5 (152–2000)	170 (50–1130)	9.2 (3.7–61)
Female	633 (149–2000)	135.5 (50–1404)	7.6 (3.7–33.3)
	*p = 0*.02*	*p<0*.001*	*p<0*.001*
Prior Treatment History			
Previously-treated	594(149–2000)	149.5 (50–1404)	8.4 (3.7–61)
Newly-diagnosed	574 (202–2000)	133 (50–974)	8.4 (4.1–21.9)
	*p = 0*.*87*	*p = 0*.04*	*p = 0*.*60*
Cancer Type			
Breast	628 (155–2000)	128.5 (50–316)	7.7 (3.7–20.7)
Colorectal	575 (149–2000)	166 (50–1404)	8.8 (3.8–61)
Lung	658 (304–2000)	143 (53–343)	8.2 (4.9–20.2)
Prostate	512 (237–2000)	170 (59–406)	9.1 (5.6–18.7)
Pancreas	602 (243–2000)	182 (97–1130)	8.8 (5.4–20.2)
Others	550 (152–2000)	150.5 (54–974)	8.5 (3.7–33.3)
	*p = 0*.04*	*p = 0*.002*	*p = 0*.*09*
Diabetes			
Yes	646.5 (149–2000)	174.5 (50–325)	9.1 (4.3–20.7)
No	577.5 (152–2000)	142.5 (50–1404)	8.3 (3.7–61)
	*p = 0*.*58*	*p = 0*.*10*	*p = 0*.*10*
Serum Creatinine			
High (>1.2 mg/dl)	417.5 (287–1921)	231 (110–701)	12.5 (3.7–33.3)
Normal (< = 1.2 mg/dl)	592 (149–2000)	143 (50–1404)	8.2 (3.7–61)
	*p = 0*.02*	*p<0*.001*	*p<0*.001*
eGFR			
Low (<60 ml/min/1.73m^2^)	381.5 (262–2000)	221 (118–701)	11.6 (3.7–33.3)
Normal (> = 60 ml/min/1.73m^2^)	601 (149–2000)	142 (50–1404)	8.2 (3.7–61)
	*p = 0*.03*	*p<0*.001*	*p<0*.001*
Subjective Global Assessment			
Malnourished	600 (149–2000)	170 (53–1404)	9.1 (4.1–33.3)
Well-nourished	581 (155–2000)	141 (50–1130)	8.2 (3.7–61)
	*p = 0*.*54*	*p = 0*.004*	*p = 0*.*06*

*p<0.05

(mg/dl = milligrams per deciliter, pg/mL = picogram per milliliter, nmol/L = nanomoles per liter, μmol/L = micromoles per liter, MMA = Methylmalonic Acid, HC = Homocysteine; eGFR = estimated glomerular filtration rate).

Of 316 patients, 5 (1.6%) were vitamin B-12 deficient based on serum vitamin B-12 <200 pg/mL, 28 (8.9%) were vitamin B-12 deficient based on serum vitamin B-12 levels <300pg/mL, 34 (10.8%) were deficient based on serum MMA levels >260 nmol/L while 55 (17.4%) were deficient based on serum HC levels >12 μmol/L. Clearly, using MMA and HC as indicators of vitamin B-12 deficiency led to a greater number of people characterized as vitamin B-12 deficient.

### Relationship between Vitamin B-12, MMA and HC

**Table 3**displays the Spearman’s rho correlation analysis between vitamin B-12, MMA and HC in the entire patient population as well as stratified across age, gender and serum creatinine. Correlation analysis revealed a significant weak negative correlation between vitamin B-12 and MMA (rho = -0.22) as well as B-12 and HC (rho = -0.35) in the total population. In males, no correlation was found between vitamin B-12 and MMA, while in females, the correlations between the parameters were similar to those observed in the total population. In patients <54 years, no correlation was found between vitamin B-12 and MMA, while in patients > = 54 years, a significant moderate negative correlation was found between vitamin B-12 and HC (rho = -0.49). In patients with normal creatinine levels, the correlations were similar to those observed in the total population, while in patients with high creatinine levels, a significant moderate negative correlation was found between vitamin B-12 and MMA (rho = -0.47). When stratified by cancer type, the only significant correlations were those between vitamin B-12 and MMA (rho = -0.26) in breast cancer, as well as vitamin B-12 and MMA (rho = -0.43) and vitamin B-12 and HC (rho = -0.57) in colorectal cancer.

**Table 3:** Spearman's rho Correlation Analysis of Vitamin B-12, MMA and HC.

	B-12	MMA	Homocysteine
*Total Population*			
B-12	1.0	-.22**	-.35**
MMA		1.0	0.41**
Homocysteine			1.0
*Stratified by Age*			
**< Median of 54 years (n = 154)**			
B-12	1.0	-.04	-.21**
MMA		1.0	.25**
Homocysteine			1.0
**> = Median of 54 years (n = 162)**			
B-12	1.0	-.36**	-.49**
MMA		1.0	.49**
Homocysteine			1.0
*Stratified by Gender*			
**Males (n = 134)**			
B-12	1.0	-.02	-.35**
MMA		1.0	.26**
Homocysteine			1.0
**Females (n = 182)**			
B-12	1.0	-.29**	-.28**
MMA		1.0	.44**
Homocysteine			1.0
*Stratified by Serum Creatinine*			
**Normal Creatinine (n = 298)**			
B-12	1.0	-.19**	-.32**
MMA		1.0	.39^*^
Homocysteine			1.0
**High Creatinine (n = 18)**			
B-12	1.0	-.47^*^	-.22
MMA		1.0	.29
Homocysteine			1.0
*Stratified by eGFR*			
**Low eGFR (n = 22)**			
B-12	1.0	-.52**	-.27
MMA		1.0	.32
Homocysteine			1.0
**Normal eGFR (n = 294)**			
B-12	1.0	-.17**	-.33**
MMA		1.0	.38**
Homocysteine			1.0

*p<0.05

**p<0.01

(MMA = Methylmalonic Acid; eGFR = estimated glomerular filtration rate).

**Table 4**provides a cross-tabulation analysis between vitamin B-12 and MMA as well as vitamin B-12 and HC using all 3 parameters as categorical variables. Of 288 patients with “sufficient” (> = 300 pg/mL) vitamin B-12 levels, 27 (9.4%) had high MMA levels, 45 (15.6%) had high HC levels, 63 (21.9%) had either high MMA or high HC levels, while 9 (3.1%) had high levels of both MMA and HC suggesting a vitamin B-12 deficient state. These results suggest that using vitamin B-12 as the sole criterion for assessing B-12 deficiency can lead to significant under-diagnosis. Adding MMA and HC to the testing panel can identify a greater number of individuals with potential B-12 deficiency.

**Table 4:** Cross-Tabulation Analysis of Vitamin B-12 with MMA and HC.

	**Vitamin B-12**		
**MMA** (*nmol/L)*	*<300 pg/mL*	*> = 300 pg/mL*	P-value
*>260*	7 (25%)	27 (9.4%)	
*< = 260*	21 (75%)	261 (90.6%)	
**Total**	**28**	**288**	0.01*
	**Vitamin B-12**		
**HC** (*μmol/L)*	*<300 pg/mL*	*> = 300 pg/mL*	
*>12*	10 (35.7%)	45 (15.6%)	
*< = 12*	18 (64.3%)	243 (84.4%)	
**Total**	**28**	**288**	0.007*
	**Vitamin B-12**		
**MMA and HC**	*<300 pg/mL*	*> = 300 pg/mL*	
*MMA>260 or HC >12*	0 (0%)	63 (21.9%)	
*MMA < = 260 or HC < = 12*	28 (100%)	225 (78.1%)	
**Total**	**28**	**288**	0.006*

*p<0.05

Numbers in parentheses are column percentages

(pg/mL = picogram per milliliter, nmol/L = nanomoles per liter, μmol/L = micromoles per liter, MMA = Methylmalonic Acid, HC = Homocysteine).

The eGFR of patients with sufficient vitamin B-12 levels but high MMA levels (n = 27) was explored to determine if low eGFR (<60 ml/min/1.73m^2^) could have led to an elevation of MMA levels. Only 5 out of 27 patients had low eGFR suggesting that high MMA levels in majority of these apparently normal B-12 patients were most likely due to underlying vitamin B-12 deficiency. Similarly, of 45 patients with sufficient vitamin B-12 levels but high HC levels, only 10 had low eGFR, once again pointing to the potential role of vitamin B-12 deficiency in causing HC elevation.

### Diagnostic Accuracy of Serum Vitamin B-12, MMA and HC in Predicting cB12

**Fig 1:**
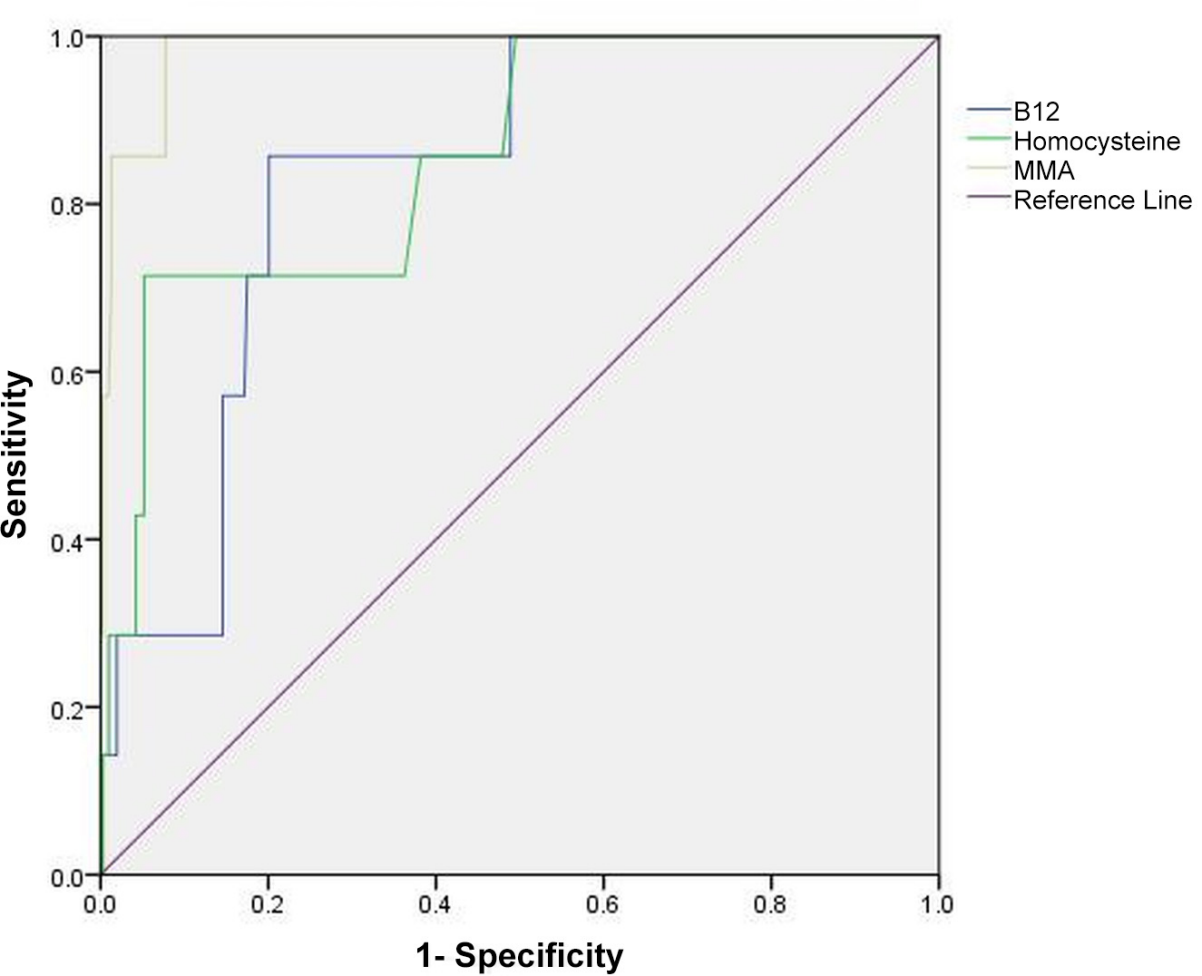
ROC curves showing the diagnostic value of vitamin B-12, MMA and HC in predicting cB12.

**Fig 1**shows the ROC curves for vitamin B-12, MMA and HC in predicting vitamin B-12 deficiency defined as a cB12 value of < −0.5 point. The sample size available for ROC analyses was 223 after excluding patients with high serum creatinine (>1.2 mg/dl), malnourished patients and patients with low eGFR (<60 ml/min/1.73m^2^). The AUCs for B-12, MMA and HC in predicting cB12 were 0.83, 0.98 and 0.85 respectively. The AUC for MMA was statistically significantly greater than that for B-12 (p = 0.02). No other pairwise comparisons were significantly different from each other. A B-12 cut-off level of 385 pg/mL provided 86% sensitivity and 80% specificity to detect B-12 deficiency. An MMA cut-off level of 413.5 nmol/L provided 86% sensitivity and 99% specificity to detect B-12 deficiency. Finally, an HC cut-off level of 15.5 μmol/L provided 71% sensitivity and 95% specificity to detect B-12 deficiency.

## Discussion

An early diagnosis of vitamin B-12 deficiency is essential in cancer patients at risk of developing CIPN because neurological damage could be irreversible and possibly be prevented by vitamin B-12 substitution. There is no well-accepted “gold standard” for confirming vitamin B-12 deficiency, and the methods to assess vitamin B-12 status fall broadly into two categories. In one, vitamin B-12 is measured directly in the blood and in the other, metabolites that accumulate as a result of the vitamin B-12 deficiencies are measured [8;28].

Measurement of serum vitamin B-12 concentration has been the mainstay for assessing suspected cases of vitamin B-12 deficiency, especially when the available resources are limited. However, there are major limitations with this approach. Serum vitamin B-12 concentrations are directly altered by the concentrations of the binding proteins. Falsely increased values are caused by myeloproliferative disorders while falsely low values can be seen with folate deficiency, pregnancy, myelomatosis, and transcobalamin deficiencies [9]. As a result, some groups now recognize cB12 as the “gold standard” to diagnose vitamin B-12 deficiency [25;26]. As described earlier, cB12 is a new approach developed and validated by Fedosov et al. that combines four B-12 biomarkers [B-12, MMA, HC and holotranscobalamin (holoTC)] into one combined measure thereby improving the precision of B-12 deficiency diagnosis.

In our current study, we evaluated the relationship of serum levels of vitamin B-12 with serum MMA and HC in cancer. There are 2 key findings of our study. First, the prevalence of vitamin B-12 deficiency in cancer is a function of the tests used to diagnose it along with their corresponding cut-offs. If vitamin B-12 deficiency is defined as serum B-12 levels < 200 pg/mL, the prevalence rate of deficiency was 1.6%. Using a B-12 cut-off point <300 pg/mL for subclinical deficiency [8], the prevalence rate was 8.9%. Finally, using MMA and HC levels, the respective prevalence rates were 10.8% and 17.4%. These findings suggest that using vitamin B-12 testing alone can lead to an under-diagnosis of the problem by up to 16%. The drastic impact that diverse cut-off selection can have on the diagnosis of vitamin B-12 deficiency has been reported previously in the literature [14;29].

The second key finding of our study is the weak association of vitamin B-12 with MMA and HC in cancer. There were 27 patients (8.5% of the total sample) who were classified as vitamin B-12 deficient by MMA but vitamin B-12 sufficient using a B-12 cut-off of 300 pg/mL. Using serum MMA in conjunction with serum vitamin B-12 would have led to the characterization of these patients as B-12 deficient resulting into an overall B-12 deficiency prevalence rate of 17.4% (versus 8.9% from using B-12 alone). This group of 27 patients in our study reflects the superior sensitivity of MMA compared to vitamin B-12 in mild vitamin B-12 deficiency. This finding was further corroborated by the observation that MMA demonstrated the best diagnostic accuracy in predicting B-12 deficiency using cB12 as the “gold standard”.

The choice of the threshold vitamin B-12 concentration for triggering follow-up is controversial. If the lower cut-off of 200 pg/mL is used, multiple patients with increased MMA and HC would be missed. If higher values, such as 500 pg/mL, are used, the majority of the patients having vitamin B-12 tests would require follow-up MMA or HC tests [9]. Given that individual tests lack enough sensitivity and specificity, we recommend that testing strategies for vitamin B-12 deficiency include B-12, MMA and HC levels used in combination in patients suspected to have risk factors for vitamin B-12 deficiency as well as in patients in whom a definitive diagnosis of vitamin B-12 deficiency cannot be reached. Recommending serum MMA as a routine test in all cancer patients to diagnose vitamin B-12 deficiency should be delayed to a point until more inexpensive methods of its determination become available.

However, one should use caution in interpreting the results of these tests against the backdrop of patients’ clinical history, physical examination and presence of other co-morbidities. This is because elevated plasma concentrations of MMA or HC can be found in patients with diabetes and renal dysfunction [22]. Other causes that can cause elevation of MMA are states of dehydration, inherited methylmalonic aciduria, and small-bowel overgrowth with bacteria producing high amounts of propionic acid, the precursor of MMA [30]. Other conditions that can cause elevations of HC are folate deficiency, hypothyroidism, vitamin B-6 deficiency, proliferative disorders and response to certain drugs [30]. Once these confounding causes can be ruled out, elevation of plasma MMA or HC can provide a sensitive indication of vitamin B-12 deficiency [8].

This study emphasizes the importance of testing for MMA and HC levels in patients with solid tumors. Detecting and treating subclinical vitamin B-12 deficiency, particularly in patients who are likely to receive neurotoxic chemotherapeutic drugs, may reduce the incidence of CIPN and allow us to use these drugs for a longer period of time. Future prospective randomized studies should evaluate the impact of vitamin B-12 supplementation in reducing the incidence of CIPN in patients receiving neurotoxic drugs. Objective evaluation using nerve conduction studies will be necessary to prove this hypothesis.

The accurate burden of vitamin B-12 deficiency in cancer can only be estimated when consensus is reached on the appropriate cut-offs to be used for different biomarkers of vitamin B-12 deficiency. This is an important area of investigation for future studies. Prospective clinical studies are also needed to investigate the impact of vitamin B-12 supplementation on these biomarkers as well as clinical outcomes such as tumor response, survival and quality of life in large cancer patient populations.

There are some limitations of this study which require careful acknowledgment. The main limitation is the cross-sectional design which cannot establish causality among the different biomarkers evaluated in the study. No formal sample size calculation was conducted before undertaking this study. We did not have data on the folate status of our patients. There is no consensus in the literature on the appropriate cut-offs to be used for assessing vitamin B-12, MMA and HC status, and this study does not help address that gap. Given that a majority of our patients were newly-diagnosed, non-diabetic, well-nourished with normal renal function, the results of this study might not be generalizable to patients with recurrent cancer including those with additional co-morbidities such as diabetes and impaired renal function. While studies in these additional patient populations are warranted, we have no reason to believe that the correlations observed among B-12, MMA and HC would be significantly different in these populations.

The strengths of this study are that all indicators were tested on all patients with no missing data, and the sample size was large enough to give adequate power for correlation and ROC analysis. Several confounding variables such as age, gender, renal function and nutritional status were taken into account while analyzing the relationship between different vitamin B-12 indicators. By using a consecutive case series of all eligible patients seen at our institution during a fixed time period, we minimized the possibility of selection bias in our study. To the best of our knowledge, this is the first study to report on the association of serum vitamin B-12 with serum MMA and HC in a large sample of cancer patients.

## Conclusion

In conclusion, using serum vitamin B-12 alone to evaluate B-12 status in cancer may fail to identify those with functional deficiency. A thorough clinical assessment is important to identify patients that may have risk factors and/or symptoms suggestive of deficiency. These patients should have additional testing of MMA and HC regardless of their B-12 levels.
